# A machine learning model to predict the need for conversion of operative approach in patients undergoing colectomy for neoplasm

**DOI:** 10.1002/cnr2.1917

**Published:** 2023-10-26

**Authors:** Keegan Guidolin, Deanna Ng, Anudari Zorigtbaatar, Sami Chadi, Fayez Quereshy

**Affiliations:** ^1^ Department of Surgery University of Toronto Toronto Ontario Canada; ^2^ Institute of Biomedical Engineering University of Toronto Toronto Ontario Canada; ^3^ Department of Surgery University Health Network Toronto Ontario Canada; ^4^ Institute of Medical Science University of Toronto Toronto Ontario Canada; ^5^ Department of Surgery Mount Sinai Hospital Toronto Ontario Canada

**Keywords:** colectomy, colorectal cancer, conversion, machine learning, surgical approach

## Abstract

**Background:**

Studies comparing conversion from laparoscopic to open approaches to colectomy have found an association between conversion and morbidity, mortality, and length of stay, suggesting that certain patients may benefit from an open approach “up‐front.”

**Aim:**

The objective of this study was to use machine learning algorithms to develop a model enabling the prediction of which patients are likely to require conversion.

**Methods and Results:**

We used ACS NSQIP data to identify patients undergoing colectomy (2014‐2019). We included patients undergoing elective colectomy for colorectal neoplasm via a minimally invasive approach or a converted approach. The outcome of interest was conversion. Variables were included in the model based on their correlation with conversion by logistic regression (*p* < .05). Two models were used: weighted logistic regression with regularization, and Random Forest classifier. The data was randomly split into training (70%) and test (30%) cohorts, and prediction performance was calculated. 24 327 cases were included (17 028 training, 7299 test). When applied to the test cohort, the models had an accuracy of 0.675 (range 0.65–0.70) in predicting conversion; c‐index ranged from 0.62–0.63. This machine learning model achieved a moderate area under the curve and a high negative predictive value, but a low positive predictive value; therefore, this model can predict (with 95% accuracy) whether a colectomy for neoplasm can be successfully completed using a minimally invasive approach.

**Conclusion:**

This model can be used to reassure surgeons of the appropriateness of a minimally invasive approach when planning for an elective colectomy.

## 1 INTRODUCTION

Colorectal surgery is a rapidly evolving field with a history of leadership and pioneering in the adoption of new technologies, most prominently minimally invasive surgery (MIS). Laparoscopic surgery is now considered the standard approach for most elective colectomies. Numerous studies have evaluated its outcomes compared to open surgery when treating colorectal cancer patients and showed short‐term and long‐term benefits such as lower mortality and morbidity, decreased length of stay and lower cost, without compromising oncological outcomes.^1^, ^2^, ^3^

Intraoperative conversion from laparoscopic to open colectomy is variably reported in the literature as occurring in 5.2%–77% of cases.^4^, ^5^ The decision to convert may be for technical reasons, like the existence of adhesions, tumor invasion into adjacent structures, and uncontrollable hemorrhage, among other factors.^6^, ^7^ Several studies compared the outcomes of conversion from laparoscopic to open colorectal resection and found increased morbidity, mortality, and length of stay; however, some recent analyses call this into question.^8^, ^9^, ^10^, ^11^, ^12^ When compared to planned open resection, conversion surgeries do not lead to worse outcomes except for a higher risk of wound infection, suggesting that some patients may have benefitted from upfront open surgery.^13^ The interpretation of these studies remains challenging given underlying selection biases between patients undergoing upfront open surgery and patients selected for laparoscopy. Previous studies have identified risk factors for conversion: age >50 years, especially >80 years; underweight, or obese; American Society of Anesthesiologists (ASA) class III–IV; a history of smoking; and weight loss were at a higher risk for conversion.^14^

The identification of surgical patients at high risk for conversion may improve shared decision‐making and patient selection for elective colectomies. In this study, we sought to identify patient‐specific and disease‐specific factors that can estimate the risk of conversion from laparoscopic to open colectomy among colorectal cancer patients undergoing an elective colectomy. We aimed to test the ability of two machine learning algorithms to predict for conversion.

## 2 MATERIALS AND METHODS

### 2.1 Data source and study population

Data were obtained through the American College of Surgeons National Surgical Quality Improvement Program (NSQIP), an international validated, quality improvement program that collects demographic, operative, and 30‐day outcome data from participating hospitals in patients undergoing specific surgical procedures. Since 2014, in addition to the general Participant User File (PUF), NSQIP has collected additional clinically relevant data on patients undergoing colectomy procedures and compiled it in the Targeted Colectomy File (TCF). The target population for this study was patients undergoing a colectomy procedure for benign or malignant neoplasm of the colon between 2014 and 2019 (inclusive).

### 2.2 Study design and cohort build

**FIGURE 1:**
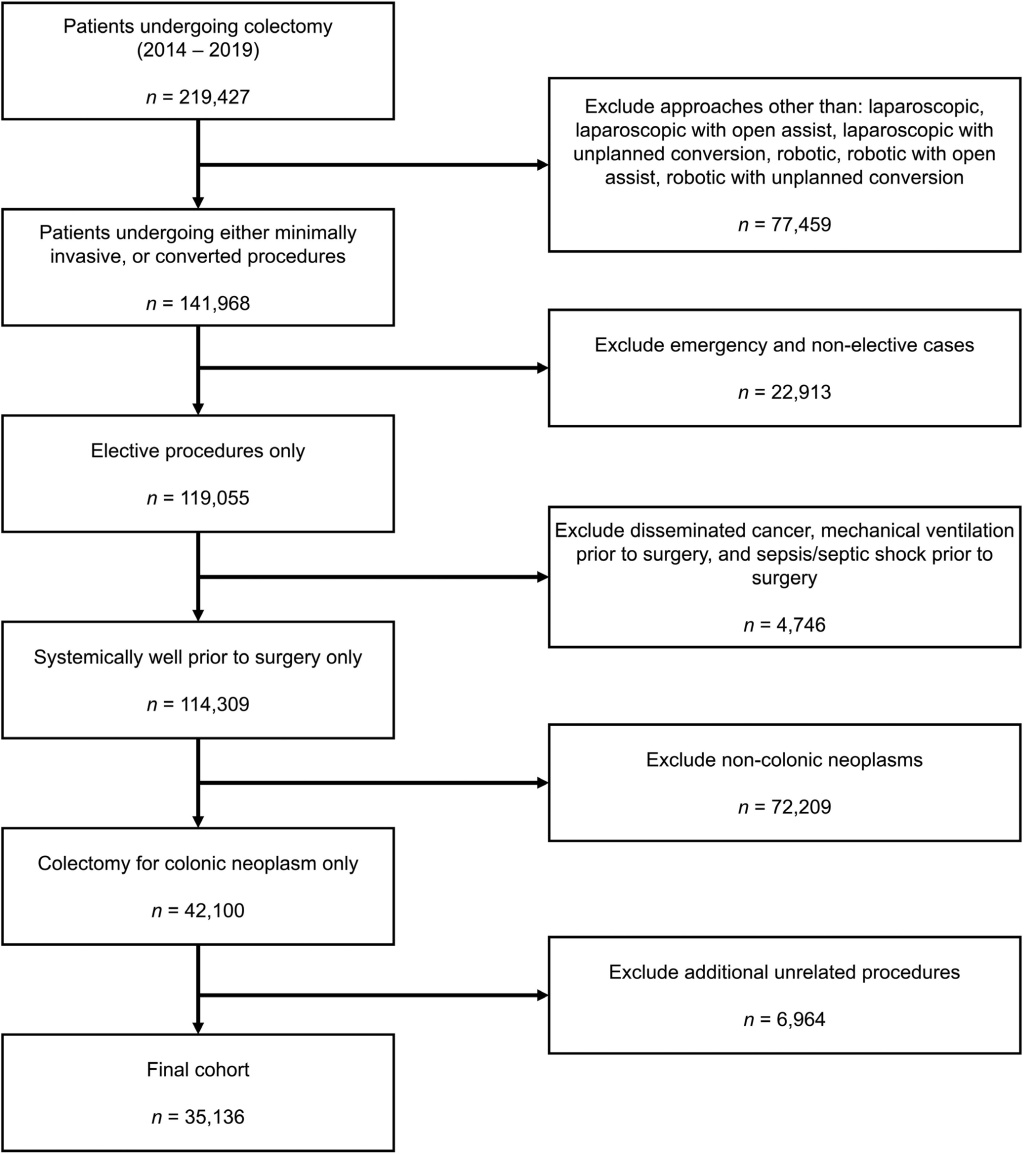
PRISMA flow diagram of cohort build. *Post‐hoc* exclusion of cases missing T‐stage data is not captured in this figure.

We performed a retrospective cohort study using the NSQIP dataset. The study cohort was built by matching records from the PUF with those from the TCF using a unique NSQIP identifier. Exclusion criteria included: patients undergoing surgery via a planned open approach, patients undergoing emergency or non‐elective surgery, patients who were systemically unwell at the time of surgery (i.e., had disseminated cancer, were mechanically ventilated, or who had sepsis or septic shock), patients who were undergoing colectomy for indications other than colonic neoplasm (included diagnoses can be found in Table S1), and patients undergoing significant additional procedures at the time of surgery (e.g., multivisceral resections, Table S2). A flow diagram of the cohort construction is shown in Figure 1.

The primary outcome of the study was any conversion from a minimally invasive approach to an open approach. Operative approach dichotomized as either MIS or converted, with laparoscopic and robotic approaches, as well as laparoscopic and robotic approaches with open assist all grouped under the umbrella MIS approach. The cohort was divided into two groups by approach (i.e., MIS and converted) and factors of interest were compared to identify significant predictors of conversion. Factors of interest included patient‐ and disease‐specific variables.

### 2.3 Statistical analysis and machine learning model

The outcome was conversion from MIS to open surgery, intraoperatively. Association with conversion screening was performed based on testing the correlation of each factor to the occurrence of conversion. This was performed using a logistic regression model. All factors with a *p*‐value <.05 were used as candidate predictors to build the predictive models (i.e., univariable regression was used to select which factors to include in subsequent predictive models).

A total of two predictive models were used: (1) weighted logistic regression with regularization and (2) Random Forest classifier. For all models, the data set was randomly split into a training cohort (70%) and a test cohort (30%). K‐fold cross validation was used to validate both models. For the Random Forest Classifier, features used were: {‘class_weight’: ‘balanced’, ‘criterion’: ‘gini’, ‘max_depth’: 2, ‘max_features’: ‘log2’, ‘min_samples_split’: 50, ‘n_estimators’: 200, ‘n_jobs’:6}.

A grid search for each randomly chosen combination of parameters was set up to identify the best tuning parameters. The different parameters set in the grid was assessed, and the optimal parameters were selected according to a maximum area under the curve (AUC). A final model was obtained by retraining the algorithm on the entire development set using the optimal parameters selected, and the performance of this final model was assessed on the testing cohort.

We measured each models' prediction performance by computing C statistics, accuracy, sensitivity and specificity. All analysis was performed using R 3.6.1 (glmnet package) and Python 3.9 (scikitlearn package).

### 2.4 Research ethics

This study was exempt from Institutional Research Ethics Board review given the use of an anonymized, retrospective, population‐based dataset that has been previously published (https://www.uhnresearch.ca/content/faq-14). It was approved by the American College of Surgeons National Surgical Quality Improvement Program.

## 3 RESULTS

A total of 35 136 patients were included. In 10 771 patients, T stage data was missing—these cases were excluded post hoc. On univariable logistic regression analysis, age, body mass index (BMI), sex, dyspnea, diabetes, ASA class, wound class, hypertension, renal failure, chronic obstructive pulmonary disease (COPD), presence of ascites, T stage, weight loss, pneumonia, steroid use, and presence of a bleeding disorder were significant predictors of conversion (Table 1).

**TABLE 1:** Univariable logistic regression for operative conversion.

Variable	Total (*n* = 24 365)	Converted (*n* = 1598)	MIS (*n* = 22 767)	Odds ratio (95% CI)	*p*‐Value
Age	68 (59–77)	70 (61–78)	68 (59–77)	1.0006 (1.0004–1.0008)	<.0001
BMI	28 (24–32)	30 (25–34)	28 (24–32)	1.039 (1.033–1.045)	<.0001
Sex					<.0001
Female	12 227	710	11 517	*Reference*	
Male	12 138	888	11 250	1.35 (1.24–1.48)	
Diabetes					<.0001
None	19 386	1167	18 219	*Reference*	
Non‐insulin dependent	3476	279	3197	1.40 (1.25–1.58)	
Insulin dependent	1503	152	1351	1.77 (1.51–2.06)	
Smoking status					.410
Non‐smoker	21 807	1410	20 397	*Reference*	
Smoker	2558	188	2370	1.06 (0.92–1.20)	
Dyspnea					<.0001
None	22 346	1433	20 913	*Reference*	
Moderate exertion	1935	154	1781	1.33 (1.14–1.55)	
At rest	84	11	73	2.21 (1.18–3.81)	
Functional status					.072
Independent	23 889	1558	22 331	*Reference*	
Partially dependent	355	34	321	1.50 (1.05–2.08)	
Totally dependent	45	2	43	0.90 (0.22–2.45)	
Wound Class					<.0001
Class I	243	14	229	*Reference*	
Class II	22 765	1419	21 346	0.94 (0.63–1.48)	
Class III	1184	122	1062	1.70 (1.10–2.74)	
Class IV	173	43	130	4.38 (2.62–7.55)	
ASA Class					<.0001
Class I	386	18	368	*Reference*	
Class II	9173	443	8730	1.31 (0.88–2.05)	
Class III	13 431	1014	12 417	2.14 (1.44–3.34)	
Class IV	1334	123	1211	2.75 (1.79–4.42)	
Hypertension	13 671	1007	12 664	1.38 (1.27–1.52)	<.0001
Renal failure	20	3	17	3.12 (1.05–7.49)	.0205
Dialysis	102	6	96	0.948 (0.47–1.70)	.870
COPD	1189	99	1090	1.34 (1.11–1.59)	.0016
Ascites	34	9	25	5.20 (2.41–10.29)	<.0001
T‐stage					<.0001
Tis	421	21	400	*Reference*	
T1	3695	189	3506	1.03 (0.67–1.66)	
T2	4729	239	4490	1.01 (0.67–1.63)	
T3	12 801	875	11 926	1.37 (0.91–2.18)	
T4	2719	274	2445	2.02 (1.33–3.25)	
CHF	213	12	201	1.04 (0.61–1.64)	.885
Steroid use	720	61	659	1.33 (1.05–1.67)	.015
Weight loss	699	67	632	1.59 (1.23–2.02)	<.001
Bleeding disorder	570	54	516	1.53 (1.18–1.95)	<.001
Transfusion	180	15	165	1.42 (0.81–2.29)	.183
Superficial SSI present at the time of surgery	2	1	1	7.78 (0.36–81.24)	.094
Pneumonia present at the time of surgery	8	3	5	5.84 (1.28–20.20)	<.001
UTI present at the time of surgery	19	1	18	0.62 (0.035–2.93)	.641

Abbreviations: ASA, American Society of Anesthesiologists; BMI, body mass index; CHF, congestive heart failure; CI, confidence interval; COPD, chronic obstructive pulmonary disease; SSI, surgical site infection; UTI, urinary tract infection.

On multivariable analysis, age, BMI, sex, diabetes, ASA class, wound class, ascites, T stage, weight loss, pneumonia remained significant predictors of conversion (Table 2).

**TABLE 2:** Multivariable logistic regression for operative conversion.

Variable	Odds ratio (95% CI)	*p*‐Value
Age	1.000 (1.000–1.001)	.0006
BMI	1.002 (1.002–1.003)	<.0001
Sex		<.0001
Female	*Reference*	
Male	1.013 (1.007–1.019)	
Dyspnea	1.003 (0.992–1.015)	.6028
Diabetes		.0005
None	*Reference*	
Non‐insulin dependent	1.008 (0.998–1.017)	
Insulin dependent	1.024 (1.01–1.037)	
ASA Class		.0003
Class I	*Reference*	
Class II	0.993 (0.968–1.018)	
Class III	1.007 (0.982–1.033)	
Class IV	1.013 (0.984–1.042)	
Class V	0.944 (0.714–1.247)	
Wound Class		<.0001
Class I	*Reference*	
Class II	1.003 (0.973–1.035)	
Class III	1.042 (1.007–1.077)	
Class IV	1.199 (1.143–1.259)	
Hypertension	1.001 (0.994–1.008)	.8138
Renal failure	1.064 (0.950–1.192)	.2332
COPD	1.006 (0.991–1.021)	.3982
Ascites	1.187 (1.093–1.290)	<.0001
T‐stage		<.0001
Tis	*Reference*	
T1	1.004 (0.979–1.029)	
T2	1.002 (0.977–1.026)	
T3	1.020 (0.996–1.045)	
T4	1.053 (1.027–1.080)	
Weight loss	1.027 (1.007–1.046)	.0004
Pneumonia present at the time of surgery	1.036 (1.145–1.610)	.0005
Bleeding disorder	1.016 (0.995–1.037)	.1372
Steroid use	1.015 (0.997–1.034)	.1039

Abbreviations: ASA, American Society of Anesthesiologists; BMI, body mass index; CI, confidence interval; COPD, chronic obstructive pulmonary disease.

**FIGURE 2:**
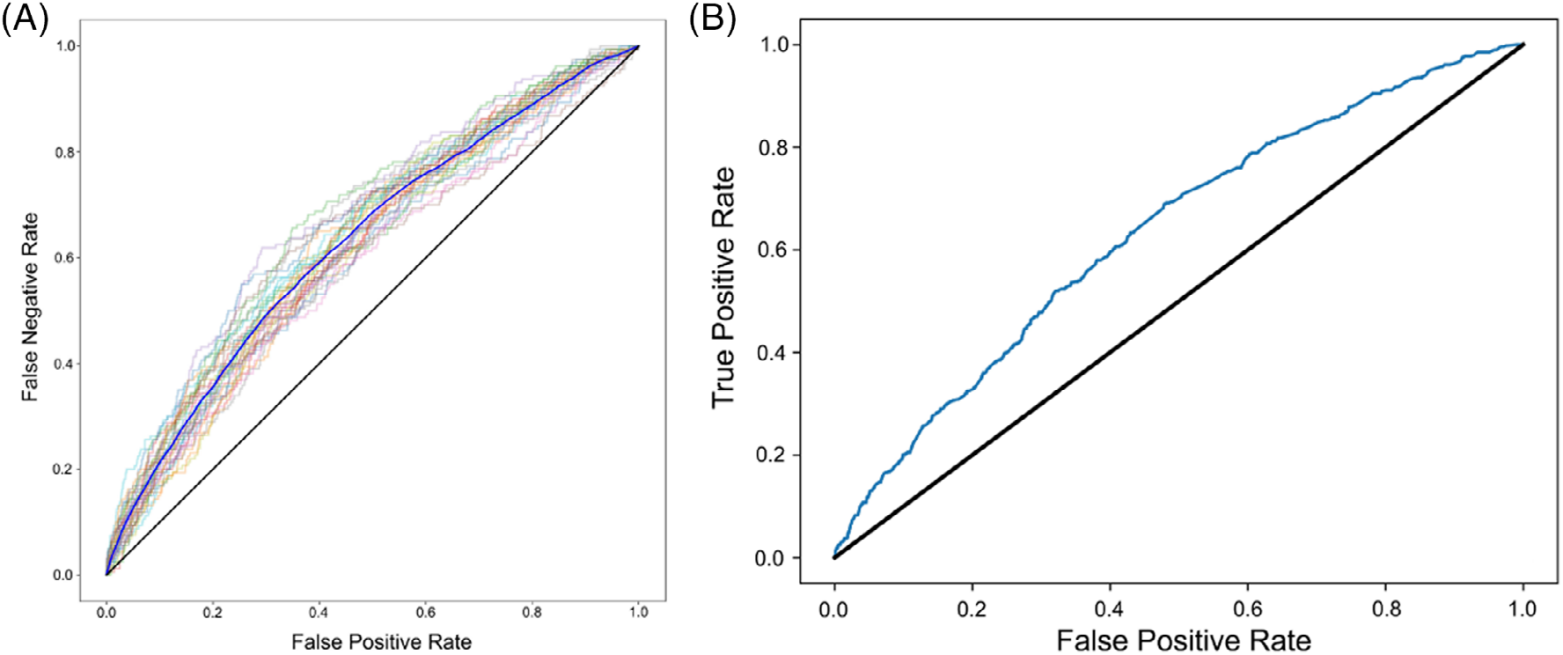
(A) Area under the curve (AUC) graph of the performance of the logistic regression machine learning model. Faded lines represent iterations of the predictive model (30 in total), the dark blue line represents the mean AUC of all iterations. Solid black line represents the reference *y* = *x* line. (B) Area under the curve (AUC) graph of the performance of the random forest classifier machine learning model. Solid black line represents the reference *y* = *x* line.

In order to develop an instrument to predict the occurrence of conversion, 24 327 patients were included (patients with missing information were excluded): 17028 (70%) in the training cohort used to create the predictive model, and 7299 (30%) patients were included in the test cohort to validate the model using only available data. Patients with missing variables were excluded from analysis. Following analysis of association with conversion status in the training cohort, variables included in the machine‐learning based models were age, BMI, sex, dyspnea, diabetes, ASA class, wound class, hypertension, renal failure, COPD, presence of ascites, T stage, weight loss, pneumonia, steroid use, and presence of a bleeding disorder. When applied to the test cohort, the models had an overall accuracy of 0.675 (range 0.65–0.70) for predicting conversion to open surgery and c‐index ranged from 0.62 to 0.63 (Figure 2).

The logistic regression model and the random forest classifier model performed similarly, with c‐indices of 0.63 and 0.62, respectively. The logistic regression model was slightly more accurate than the random forest classifier model (0.70 vs. 0.65) and had a higher specificity (0.72 vs. 0.65) but a lower sensitivity (0.48 vs. 0.53). The positive predictive values (PPV) for both models were low (0.11 and 0.10), but negative predictive values (NPV) for both models were high (0.95), see Table 3.

**TABLE 3:** Performance metrics for two machine learning models for the prediction of conversion of operative approach.

Model	C‐index	Accuracy	PPV	NPV	Sensitivity	Specificity	F1 Score
Logistic regression	0.633	0.70	0.11	0.95	0.48	0.72	0.55
Random forest classifier	0.616	0.65	0.10	0.95	0.53	0.65	0.56

Abbreviations: NPV, negative predictive value; PPV, positive predictive value.

## 4 DISCUSSION

This study presents two machine learning models to predict conversion of operative approach in colectomy (from laparoscopic to open), using a large international dataset collected through NSQIP. The two models demonstrated similar performance across metrics, suggesting that neither approach was superior in this context.

The performance analysis of these models demonstrates a moderate AUC, with a high NPV and a low PPV. This suggests that these models can provide a high degree of confidence as to whether a minimally invasive procedure can be successfully completed; however, other clinical decision measures must be used to accurately predict which patients might require conversion. Given the numerous potential benefits associated with an MIS approach to colectomy, the model described here may provide physicians with reassurance that an MIS approach is feasible in cases of uncertainty.

Though the goal of the study was to create a model that can reliably predict which patients undergoing colectomy for neoplasia will require conversion (i.e., a model with a high PPV), we instead created a model that reliably predicts which patients *will not* require conversion. This may, in reality, be more clinically useful because it provides reassurance to patients and surgeons who are favoring an MIS approach. In contrast, a model with a high PPV may dissuade patients and surgeons from attempting an MIS colectomy. This is an undesirable position to be in, given recent evidence showing that—contrary to early research—conversion does not confer an increased risk of complications or adverse outcomes in comparison to a planned open approach, and that patients are more likely to benefit if MIS is attempted than if it is not.^12^, ^15^, ^16^, ^17^, ^18^, ^19^

Although the models used in our study did not achieve high performance metrics, other, more sophisticated machine learning models (like neural network and support vector machine models) may hold promise—this a potential future direction of study.

Our study was limited by the retrospective nature of its design, as well as its use of the NSQIP database. Though this database has been frequently used for outcomes‐based research, it is imperfect and does not capture many of the granular details that might be useful in answering a research question like this one. For example, the NSQIP database does not capture reason for operative conversion, nor the timing of operative conversion (i.e., at what point in the procedure conversion occurred). Prior research suggests that these factors can provide a more nuanced understanding of conversion by differentiating between “types of conversion.” One of the major categories traditionally discussed is the a priori likely conversions, in which the surgeon suspects that a case may be difficult to perform via MIS and decides to convert after quickly surveying the state of the abdominal anatomy. This is in contrast to a conversion that occurs later in the case following an intraoperative event (e.g., an injury) that cannot be readily controlled using an MIS approach.^20^ Similarly, the NSQIP database does not capture pertinent aspects of a patient's medical history, such as the presence of prior abdominal surgery, or the level and type of training the surgeons received. A more granular dataset providing such variables may have allowed our machine‐learning model to predict for these different types of conversion more accurately; this is an interesting direction for future research. A notable strength of this study is its large sample size and its novel approach to the question of whether or not operative conversion in cases of colectomy can be predicted in a meaningful way.

## 5 CONCLUSION

The machine‐learning model described here can be used to provide patients and surgeons with a high degree of reassurance that a colectomy can be successfully performed using an MIS approach; however, other clinical decision measures must be used to positively predict for operative conversion.

## AUTHOR CONTRIBUTIONS

**Keegan Guidolin:** Conceptualization (lead); data curation (lead); project administration (lead); writing – original draft (lead). **Deanna Ng:** Data curation (equal); formal analysis (lead); methodology (lead); software (lead); writing – review and editing (equal). **Anudari Zorigtbaatar:** Data curation (supporting); investigation (supporting); writing – review and editing (supporting). **Sami Chadi:** Conceptualization (equal); methodology (equal); project administration (equal); supervision (equal); writing – review and editing (equal). **Fayez A Quereshy:** Conceptualization (equal); methodology (equal); project administration (equal); supervision (equal); writing – review and editing (equal).

## FUNDING INFORMATION

This research did not receive any specific grant from funding agencies in the public, commercial, or not‐for‐profit sectors.

## CONFLICT OF INTEREST STATEMENT

The authors have stated explicitly that there are no conflicts of interest in connection with this article.

## ETHICS STATEMENT

This study was exempt from Institutional Research Ethics Board review given the anonymized, retrospective, population‐based dataset. It was approved by the American College of Surgeons National Surgical Quality Improvement Program

## DISCLOSURES

Drs. Keegan Guidolin, Deanna Ng, and Anudari Zorigtbaatar have no disclosures. In the past 5 years, Dr. Sami Chadi has received honoraria from Stryker Endoscopy, and Dr. Fayez Quereshy has received honoraria from Minogue Medical and Medtronic.

## PRIOR PRESENTATIONS

This work was presented at the American College of Surgeons Clinical Congress 2022 in San Diego, CA, in October 2022. Its abstract was accordingly published in the Journal of the American College of Surgeons (JACS).

## DISCLAIMER

American College of Surgeons National Surgical Quality Improvement Program and the hospitals participating in the ACS NSQIP are the source of the data used herein; they have not verified and are not responsible for the statistical validity of the data analysis or the conclusions derived by the authors.

## Supporting information


**Data S1.** Supporting Appendix.

**Table S1.** Included diagnoses and corresponding International Classification of Disease code.
**Table S2.** Included additional procedures conducted at the time of the primary surgery.

## Data Availability

The data that support the findings of this study are available from the corresponding author upon reasonable request.
