# Supplementary material for: A machine learning model to predict the need for conversion of operative approach in patients undergoing colectomy for neoplasm
Source: Cancer Rep (Hoboken). 2023 Oct 26;7(1):e1917. doi: 10.1002/cnr2.1917 (PMC10809191; doi:10.1002/cnr2.1917)
Supplement: Supplementary file 1 — Data S1. Supporting Appendix. [file CNR2-7-e1917-s002.docx]

**Appendix 2a.** Python code for random forest classifier

#!/usr/bin/env python3

# basic libraries

# first neural network with keras tutorial

from numpy import loadtxt

import pandas as pd

import numpy as np

import matplotlib.pyplot as plt

import seaborn as sns

from sklearn.model_selection import train_test_split, GridSearchCV, cross_val_score, RepeatedStratifiedKFold, StratifiedKFold

from sklearn.metrics import accuracy_score, confusion_matrix,roc_curve, roc_auc_score, precision_score, recall_score, precision_recall_curve

from sklearn.preprocessing import LabelEncoder#for train test splitting

from sklearn.model_selection import train_test_split#for decision tree object

from sklearn.tree import DecisionTreeClassifier#for checking testing results

from sklearn.metrics import classification_report, confusion_matrix#for visualizing tree

from sklearn.tree import plot_tree

#dataset

df = pd.read_excel("Conversion dataset Sep 8 to send.xlsx")

df = df.dropna()

#ns.pairplot(data=df, hue = 'Y')

#plt.show()

#print(df)

X = df.drop('Y',axis=1)

y = df['Y']

X_train, X_test, y_train, y_test = train_test_split(X , y, test_size = 0.3, random_state = 42)

print("Training split input- ", X_train.shape)

print("Testing split input- ", X_test.shape)

# Creating a function to report confusion metrics

def confusion_metrics(conf_matrix):

# save confusion matrix and slice into four pieces

TP = conf_matrix[1][1]

TN = conf_matrix[0][0]

FP = conf_matrix[0][1]

FN = conf_matrix[1][0]

print('True Positives:', TP)

print('True Negatives:', TN)

print('False Positives:', FP)

print('False Negatives:', FN)

# calculate accuracy

conf_accuracy = (float (TP+TN) / float(TP + TN + FP + FN))

# calculate mis-classification

conf_misclassification = 1- conf_accuracy

# calculate the sensitivity

conf_sensitivity = (TP / float(TP + FN))

# calculate the specificity

conf_specificity = (TN / float(TN + FP))

# calculate precision

conf_precision = (TN / float(TN + FP))

# calculate f_1 score

conf_f1 = 2 * ((conf_precision * conf_sensitivity) / (conf_precision + conf_sensitivity))

print('-'*50)

print(f'Accuracy: {round(conf_accuracy,2)}')

print(f'Mis-Classification: {round(conf_misclassification,2)}')

print(f'Sensitivity: {round(conf_sensitivity,2)}')

print(f'Specificity: {round(conf_specificity,2)}')

print(f'Precision: {round(conf_precision,2)}')

print(f'f_1 Score: {round(conf_f1,2)}')

# define model

model = DecisionTreeClassifier()

# define grid

w = [{0:1000,1:100},{0:1000,1:10}, {0:1000,1:1.0},

{0:500,1:1.0}, {0:400,1:1.0}, {0:300,1:1.0}, {0:200,1:1.0},

{0:150,1:1.0}, {0:100,1:1.0}, {0:99,1:1.0}, {0:10,1:1.0},

{0:0.01,1:1.0}, {0:0.01,1:10}, {0:0.01,1:100},

{0:0.001,1:1.0}, {0:0.005,1:1.0}, {0:1.0,1:1.0},

{0:1.0,1:0.1}, {0:10,1:0.1}, {0:100,1:0.1},

{0:10,1:0.01}, {0:1.0,1:0.01}, {0:1.0,1:0.001}, {0:1.0,1:0.005},

{0:1.0,1:10}, {0:1.0,1:99}, {0:1.0,1:100}, {0:1.0,1:150},

{0:1.0,1:200}, {0:1.0,1:300},{0:1.0,1:400},{0:1.0,1:500},

{0:1.0,1:1000}, {0:10,1:1000},{0:100,1:1000} ]

param_grid = dict(class_weight = w)

# define evaluation procedure

cv = RepeatedStratifiedKFold(n_splits=10, n_repeats=3, random_state=1)

# define grid search

grid = GridSearchCV(estimator=model, param_grid=param_grid, n_jobs=-1, cv=cv, scoring='roc_auc')

# execute the grid search

grid_result = grid.fit(X_train,y_train)

# make the predictions

y_pred = grid.predict_proba(X_test)[:,1]

y_pred = np.where(y_pred>0.5, 1, 0)

# report the best configuration

print("Best: %f using %s" % (grid_result.best_score_, grid_result.best_params_))

# confusion matrix on the test data.

print('\nConfusion matrix of Random Forest optimized for {} on the test data:')

# Creating the confusion matrix

cm = confusion_matrix(y_test, y_pred)

# Assigning columns names

cm_df = pd.DataFrame(cm,

columns = ['Predicted Negative', 'Predicted Positive'],

index = ['Actual Negative', 'Actual Positive'])

confusion_metrics(cm)

# report the best configuration

print("Best: %f using %s" % (grid_result.best_score_, grid_result.best_params_))

# report all configurations

means = grid_result.cv_results_['mean_test_score']

stds = grid_result.cv_results_['std_test_score']

params = grid_result.cv_results_['params']

for mean, stdev, param in zip(means, stds, params):

print("%f (%f) with: %r" % (mean, stdev, param))

# Defining the decision tree algorithm

dtree=DecisionTreeClassifier(class_weight={0:1, 1:12})

dtree.fit(X_train,y_train)

print('Decision Tree Classifier Created')

# Predicting the values of test data

y_pred = dtree.predict(X_test)

print("Classification report - \n", classification_report(y_test,y_pred))

cm = confusion_matrix(y_test, y_pred)

plt.figure(figsize=(5,5))

sns.heatmap(data=cm,linewidths=.5, annot=True,square = True, cmap = 'Blues')

plt.ylabel('Actual label')

plt.xlabel('Predicted label')

all_sample_title = 'Accuracy Score: {0}'

format(dtree.score(X_test, y_test))

plt.title(all_sample_title, size = 15)

plt.show()

**Appendix 2b.** Python code for logistic regression

#!/usr/bin/env python3

# basic libraries

import pandas as pd

import numpy as np

from matplotlib import pyplot as plt

import seaborn as sns

from sklearn.datasets import make_blobs, make_classification

#import keras

#from keras.models import Sequential

#from keras.layers import Dense

#from keras.wrappers.scikit_learn import KerasClassifier

#import model and matrics

from sklearn.linear_model import LogisticRegression

from sklearn.model_selection import train_test_split, GridSearchCV, cross_val_score, RepeatedStratifiedKFold, StratifiedKFold

from sklearn.metrics import accuracy_score, confusion_matrix,roc_curve, roc_auc_score, precision_score, recall_score, precision_recall_curve

from sklearn.metrics import f1_score

#dataset

df = pd.read_excel("Conversion dataset Sep 8 to send.xlsx")

df = df.dropna()

#print(df)

# check the distribution

distribution = df["Y"].value_counts()/df.shape[0]

print(distribution)

# scatter plot

plt.figure(figsize=(10,5))

#im = sns.scatterplot(data=df,x="age",y="BMI",hue="Y")

#plt.show()

#LogisticRegression

# split dataset into x,y

x = df.drop('Y',axis=1)

y = df['Y']

# train-test split

X_train, X_test, y_train, y_test = train_test_split(x, y, test_size=0.3, random_state=13)

# # define model

# lg1 = LogisticRegression(random_state=13, class_weight=None, max_iter = 1000)

# # fit it

# lg1.fit(X_train,y_train)

# # test

# y_pred = lg1.predict_proba(X_test)[:,1]

# y_pred = np.where(y_pred>0.45, 1, 0)

# # performance

# print(f'Accuracy Score: {accuracy_score(y_test,y_pred)}')

# print(f'Confusion Matrix: \n{confusion_matrix(y_test, y_pred)}')

# print(f'Area Under Curve: {roc_auc_score(y_test, y_pred)}')

# print(f'Recall score: {recall_score(y_test,y_pred)}')

# # define class weights

# w = {0:1, 1:100}

# # define model

# lg2 = LogisticRegression(random_state=13, class_weight=w, max_iter = 1000)

# # fit it

# lg2.fit(X_train,y_train)

# # test

# y_pred = lg2.predict_proba(X_test)[:,1]

# y_pred = np.where(y_pred>0.5, 1, 0)

# # performance

# print(f'Accuracy Score: {accuracy_score(y_test,y_pred)}')

# print(f'Confusion Matrix: \n{confusion_matrix(y_test, y_pred)}')

# print(f'Area Under Curve: {roc_auc_score(y_test, y_pred)}')

# print(f'Recall score: {recall_score(y_test,y_pred)}')

# # define weight hyperparameter

# w = [{0:1000,1:100},{0:1000,1:10}, {0:1000,1:1.0},

# {0:500,1:1.0}, {0:400,1:1.0}, {0:300,1:1.0}, {0:200,1:1.0},

# {0:150,1:1.0}, {0:100,1:1.0}, {0:99,1:1.0}, {0:10,1:1.0},

# {0:0.01,1:1.0}, {0:0.01,1:10}, {0:0.01,1:100},

# {0:0.001,1:1.0}, {0:0.005,1:1.0}, {0:1.0,1:1.0},

# {0:1.0,1:0.1}, {0:10,1:0.1}, {0:100,1:0.1},

# {0:10,1:0.01}, {0:1.0,1:0.01}, {0:1.0,1:0.001}, {0:1.0,1:0.005},

# {0:1.0,1:10}, {0:1.0,1:99}, {0:1.0,1:100}, {0:1.0,1:150},

# {0:1.0,1:200}, {0:1.0,1:300},{0:1.0,1:400},{0:1.0,1:500},

# {0:1.0,1:1000}, {0:10,1:1000},{0:100,1:1000} ]

# hyperparam_grid = {"class_weight": w }

# # define model

# lg3 = LogisticRegression(random_state=13, max_iter=1000)

# # define evaluation procedure

# grid = GridSearchCV(lg3,hyperparam_grid,scoring="roc_auc", cv=10, n_jobs=-1, refit=True)

# grid.fit(x,y)

# print(f'Best score: {grid.best_score_} with param: {grid.best_params_}')

# weighted logistic regression for class imbalance with heuristic weights

from numpy import mean

from sklearn.datasets import make_classification

from sklearn.model_selection import cross_val_score

from sklearn.model_selection import RepeatedStratifiedKFold

from sklearn.linear_model import LogisticRegression

# generate dataset

# define model

model = LogisticRegression(solver='lbfgs', class_weight='balanced', max_iter=1000)

# define evaluation procedure

cv = RepeatedStratifiedKFold(n_splits=10, n_repeats=3, random_state=1)

# evaluate model

scores = cross_val_score(model, X_train, y_train, scoring='roc_auc', cv=cv, n_jobs=-1)

# summarize performance

print('Mean ROC AUC: %.3f' % mean(scores))

#F1 score

model.fit(X_train,y_train)

predictions = model.predict(X_test)

print(model.score(X_test, y_test))

f1score = f1_score(y_test, predictions)

print('F1 score:', f1score)

# AUC plot; ref: https://www.kaggle.com/code/kanncaa1/roc-curve-with-k-fold-cv/notebook

from sklearn.metrics import roc_curve, auc

from scipy import interp

fig1 = plt.figure(figsize=[12,12])

tprs = []

aucs = []

mean_fpr = np.linspace(0,1,100)

i = 1

for train, test in cv.split(x,y):

prediction = model.fit(x.iloc[train],y.iloc[train]).predict_proba(x.iloc[test])

fpr, tpr, t = roc_curve(y.iloc[test], prediction[:, 1])

tprs.append(interp(mean_fpr, fpr, tpr))

roc_auc = auc(fpr, tpr)

aucs.append(roc_auc)

plt.plot(fpr, tpr, lw=2, alpha=0.3, label='ROC fold %d (AUC = %0.02f)' % (i, roc_auc))

i= i+1

mean_tpr = np.mean(tprs, axis=0)

mean_auc = auc(mean_fpr, mean_tpr)

plt.plot(mean_fpr, mean_tpr, color='blue',

label=r'Mean ROC (AUC = %0.02f )' % (mean_auc),lw=2, alpha=1)

plt.plot([0, 1], [0, 1], color = 'black', linewidth = 2)

plt.xlabel('False Positive Rate')

plt.ylabel('True Positive Rate')

plt.title('ROC')

plt.legend(loc="lower right", prop = {"size":5})

plt.savefig("lg_auc.jpg", dpi=1000)

plt.show()
