# Supplementary material for: A machine learning model to predict the need for conversion of operative approach in patients undergoing colectomy for neoplasm
Source: Cancer Rep (Hoboken). 2023 Oct 26;7(1):e1917. doi: 10.1002/cnr2.1917 (PMC10809191; doi:10.1002/cnr2.1917)
Supplement: Supplementary file 2 — Table S1. Included diagnoses and corresponding International Classification of Disease code. Table S2. Included additional procedures conducted at the time of the primary surgery. [file CNR2-7-e1917-s001.docx]

| **Diagnosis** | **ICD Code** |
| --- | --- |
| Malignant neoplasm of hepatic flexure | 153^a^ |
| Malignant neoplasm of transverse colon | 153.1^a^ |
| Malignant neoplasm of descending colon | 153.2^a^ |
| Malignant neoplasm of sigmoid colon | 153.3^a^ |
| Malignant neoplasm of cecum | 153.4^a^ |
| Malignant neoplasm of ascending colon | 153.6^a^ |
| Malignant neoplasm of splenic flexure | 153.7^a^ |
| Malignant carcinoid tumor of the cecum | 209.12^a^ |
| Malignant carcinoid tumor of the ascending colon | 209.13^a^ |
| Malignant carcinoid tumor of the transverse colon | 209.14^a^ |
| Malignant carcinoid tumor of the descending colon | 209.15^a^ |
| Malignant carcinoid tumor of the sigmoid colon | 209.16^a^ |
| Carcinoma *in situ* of the colon | 230.3^a^ |
| Malignant neoplasm of the colon | C18.0^b^ |
| Malignant neoplasm of the ascending colon | C18.2^b^ |
| Malignant neoplasm of the hepatic flexure | C18.3^b^ |
| Malignant neoplasm of the transverse colon | C18.4^b^ |
| Malignant neoplasm of the splenic flexure | C18.5^b^ |
| Malignant neoplasm of the descending colon | C18.6^b^ |
| Malignant neoplasm of the sigmoid colon | C18.7^b^ |
| Malignant carcinoid tumor of the cecum | C7A.021^b^ |
| Malignant carcinoid tumor of the ascending colon | C7A.022^b^ |
| Malignant carcinoid tumor of the transverse colon | C7A.023^b^ |
| Malignant carcinoid tumor of the descending colon | C7A.024^b^ |
| Malignant carcinoid tumor of the sigmoid colon | C7A.025^b^ |
| Carcinoma *in situ* of the colon | D01.0^b^ |
| Benign neoplasm of the cecum | D12.0^b^ |
| Benign neoplasm of the ascending colon | D12.2^b^ |
| Benign neoplasm of the transverse colon | D12.3^b^ |
| Benign neoplasm of the descending colon | D12.4^b^ |
| Benign neoplasm of the sigmoid colon | D12.5^b^ |
| Neoplasm of uncertain behaviour of the colon | D37.4^b^ |
| Polyp of the colon | K63.5^b^ |

Table S1. Included diagnoses and corresponding International Classification of Disease code. ICD = International Classification of Disease. ^a^ ICD-9 code; ^b^ ICD-10 code.

| **Procedure** | **CPT Code** |
| --- | --- |
| Abdl lmphadec reg celiac gstr portal pripncrtc | 38747 |
| Anesthesia intraperitoneal lower abd w/laps nos | 840 |
| Anoscopy dx w/collj spec br/wa spx when prfrmd | 46600 |
| Anoscopy dx w/hra &chem agnts enhancement w/bx | 46607 |
| Anoscopy w/rmvl lesion cautery | 46610 |
| Anrct xm surg req anes general spi/edrl dx | 45990 |
| Appendec indicated purpose oth major px not spx | 44955 |
| Appendectomy | 44950 |
| Artl cathj/cannulj mntr/transfusion spx prq | 36620 |
| Bx anorectal wall anal approach | 45100 |
| Closure enterostomy lg/small intestine | 44620 |
| Clsr ntrstm lg/sm rescj & anast oth/thn clrct | 44625 |
| Clsr ntrstm lg/sm rescj & colorectal anastomosis | 44626 |
| Colct tot abdl w/o prctect w/ileost/ileopxts | 44150 |
| Colectomy partial w/anastomosis | 44140 |
| Colectomy prtl w/coloproctostomy | 44145 |
| Colectomy prtl w/end colostomy & clsr dstl sgmt | 44143 |
| Colectomy prtl w/rmvl terminal ileum & ileocolos | 44160 |
| Colectomy prtl w/skin level cecost/colostomy | 44141 |
| Colectomy tot abd w/proctectomy ileoanal anast | 44157 |
| Colonoscopy flx dx w/collj spec when pfrmd | 45378 |
| Colonoscopy flx dx w/wo collj specimens | 45378 |
| Colonoscopy flx w/endoscopic mucosal resection | 45390 |
| Colonoscopy flx w/removal of foreign body(s) | 45379 |
| Colonoscopy w/biopsy single/multiple | 45380 |
| Colostomy/skin level cecostomy | 44320 |
| Colotomy exploration/biopsy/foreign body removal | 44025 |
| Colsc flexible w/control bleeding any method | 45382 |
| Colsc flx prox splenic flxr rmvl les caut | 45384 |
| Colsc flx prox splenic flxr rmvl les snare tq | 45385 |
| Colsc flx prox splenic flxr sbmcsl njx | 45381 |
| Colsc flx w/removal lesion by hot bx forceps | 45384 |
| Colsc flx w/rmvl of tumor polyp lesion snare tq | 45385 |
| Colsc flx with directed submucosal njx any sbst | 45381 |
| Corrj malrotation bands&/rdctj volvulus | 44055 |
| Dilat rct strix spx under anes oth/thn local | 45910 |
| Doc order vte prophyl w/in 24 hrs prior surg | 4044F |
| Enteroenterost anast int w/wo cutan ntrstm spx | 44130 |
| Enterolss fring intstinal adhesion spx | 44005 |
| Enterorrhaphy 1perforation | 44602 |
| Enterorrhaphy multiple perforations | 44603 |
| Enterotomy sm int oth/thn duo expl bx/fb rmvl | 44020 |
| Injection anes ilioinguinal iliohypogastric nrvs | 64425 |
| Injection anes other peripheral nerve/branch | 64450 |
| Injection anesthetic agent femoral nerve single | 64447 |
| Insj temp ndwellg bladder catheter complicated | 51703 |
| Insj temp ndwellg bladder catheter simple | 51702 |
| Intraoperative colonic lavage | 44701 |
| Intstinal stricturoplasty w/wo dilat obstrcj | 44615 |
| Laparoscopic appendectomy | 44970 |
| Laparoscopy colectomy partial w/anastomosis | 44204 |
| Laparoscopy enterolysis separate procedure | 44180 |
| Laparoscopy surg colostomy/skn lvl cecostomy | 44188 |
| Laparoscopy surg ileostomy/jejunostomy non-tube | 44187 |
| Laparoscopy surg w/bx single/multiple | 49321 |
| Laparoscopy w/lysis of adhesions | 58660 |
| Laparoscopy w/omentopexy | 49326 |
| Laps bi tot pel lmphadec & pri-aortic lymph bx 1 | 38572 |
| Laps clsr ntrstm lg/sm int w/rescj & anastomosis | 44227 |
| Laps colct ttl abd w/prctect ileoanal anastomsis | 44211 |
| Laps colectmy prtl w/colopxtstmy lw anast w/clst | 44208 |
| Laps colectomy abdl w/proctectomy w/ileostomy | 44212 |
| Laps colectomy prtl w/colopxtstmy lw anast | 44207 |
| Laps colectomy prtl w/end clst & clsr dstl sgm | 44206 |
| Laps colectomy prtl w/rmvl terminal ileum | 44205 |
| Laps colectomy tot w/o prctect w/ileost/ileopxts | 44210 |
| Laps moblj splenic flxr pfrmd w/prtl colectomy | 44213 |
| Laps surg retroperitoneal lymph node bx 1/mlt | 38570 |
| Laps w/bi tot pel lmphadec & omntc lymph bx | 38573 |
| Lmtd lmphadec staging spx pel&para-aortic | 38562 |
| Lmtd lmphadec staging spx rpr aortic&/splenic | 38564 |
| Moblj splenic flxr pfrmd conjunct w/prtl colct | 44139 |
| Ndsc eval intstinal pouch dx w/collj spec spx | 44385 |
| Neg pressure wound therapy non dme </= 50 sq cm | 97607 |
| Negative pressure wound therapy </= 50 sq cm | 97605 |
| Negative pressure wound therapy dme </= 50 sq cm | 97605 |
| Negative pressure wound therapy dme >50 sq cm | 97606 |
| Omental flap intra-abdominal | 49905 |
| Omntc epiploectomy rescj omentum spx | 49255 |
| Path consltj surg 1st blk frozen sctj 1 spec | 88331 |
| Pel lmphadec w/xtrnl iliac hypogstr&obturator | 38770 |
| Peritoneal lavage w/wo imaging guidance | 49084 |
| Placement seton | 46020 |
| Revj colostomy w/rpr paraclst hernia spx | 44346 |
| Revj ileostomy complic rcnstj in-depth spx | 44314 |
| Revj ileostomy simple rls superficial scar spx | 44312 |
| Rpr epigastric hernia incarcerated | 49572 |
| Rpr epigastric hernia reducible spx | 49570 |
| Rpr tabdl lmphadec extnsv w/pel aortic&rnl | 38780 |
| Rpr umbilical hernia age 5 yrs/> incarcerated | 49587 |
| Rpr umbilical hrna 5 yrs/> reducible | 49585 |
| Sgmdsc flx dired sbmcsl njx any sbst | 45335 |
| Sgmdsc flx rmvl tum polyp/oth les snare tq | 45338 |
| Sigmoidoscopy flx ablation tumor polyp/oth les | 45346 |
| Sigmoidoscopy flx control bleeding | 45334 |
| Sigmoidoscopy flx dx w/collj spec br/wa if pfrmd | 45330 |
| Sigmoidoscopy flx dx w/wo collj specimens | 45330 |
| Sigmoidoscopy flx w/biopsy single/multiple | 45331 |
| Sigmoidoscopy flx with with band ligation(s) | 45350 |
| Sutr lg intestine 1/mult perforat w/o colostomy | 44604 |
| Tap block bilateral by continuous infusion(s) | 64489 |
| Tap block bilateral by injection(s) | 64488 |
| Tap block unilateral by continuous infusion(s) | 64487 |
| Tap block unilateral by injection(s) | 64486 |
| Ther proph/dx njx iv push single/1st sbst/drug | 96374 |
| Transfusion blood/blood components | 36430 |
| Ultrasonic guidance intraoperative | 76998 |

Table S2. Included additional procedures conducted at the time of the primary surgery.
